# Likelihood of Accomplishing an In-Patient Hysteroscopic Myomectomy in a One-Step Procedure: A Systematic Review and Meta-Analysis

**DOI:** 10.1155/2020/4208497

**Published:** 2020-01-08

**Authors:** Ugo Indraccolo, Vittorio Bini, Alessandro Favilli

**Affiliations:** ^1^Department of Obstetrics and Gynaecology, USL Umbria 1, Alta Valle del Tevere Hospital, Città di Castello (PG), Umbria, Italy; ^2^Department of Medicine, University of Perugia, Perugia, Italy

## Abstract

**Purpose:**

To assess the feasibility rate of one-step hysteroscopic myomectomy according to the technique adopted.

**Methods:**

In July 2016, PubMed, ClinicalTrials.gov, SCOPUS, Scielo, and AJOL databases were used for searching references. Series of in-patient hysteroscopic myomectomies reporting success rate in only one-step procedure, categorization of submucous fibroids, explanation of the surgical technique, and description of patients were considered eligible for meta-analysis (retrospective, prospective randomized studies). Two authors extracted the data. Rate of myomectomies accomplished in only a surgical step and rate of intraoperative complications were extracted per protocol. A modified GRADE score was used for quality assessment. Random-effect models were already assumed. Mean rates were compared among subgroups.

**Results:**

One thousand two hundred and fifty-seven studies were screened and 241 of these were read for eligibility. Seventy-eight series were included in qualitative synthesis and 24 series were included in quantitative synthesis. Wide heterogeneity was found. In series with <50% of G2 myomas treated, the slicing technique feasibility rate was 86.5% while techniques for enucleating the deep portion of the myomas showed a feasibility rate of 92.3% (*p* < 0.001). In series with ≥50% of G2 myomas treated, the slicing technique feasibility rate was 70.6% while techniques for enucleating the deep portion of myomas showed a feasibility rate of 88.4% (*p* < 0.001). In series with ≥50% of G2 myomas treated, the slicing technique feasibility rate was 70.6% while techniques for enucleating the deep portion of myomas showed a feasibility rate of 88.4% (

**Conclusion:**

In case of submucous myomas with intramural development, the slicing technique was correlated with a lower rate of in-patient hysteroscopic myomectomies accomplished in a one-step procedure and a higher complications rate.

## 1. Introduction

Uterine myomas, also called leiomyomas or fibroids, are benign, monoclonal tumours developing from the smooth muscle cells of the myometrium. Myomas represent the most common pathology of the female genital tract causing abnormal uterine bleeding, pelvic pain, and infertility [1–3]. Although it has been estimated that the majority of uterine fibroids is asymptomatic, submucous myomas account for 5–10% of all fibroids and are correlated with the most severe symptomatology [4, 5].

The advent of endoscopic surgery has revolutionized the treatment of submucous myomas, offering a valid alternative to hysterotomy or hysterectomy. Nowadays, the resectoscopic myomectomy is considered the gold standard in the treatment of submucous myomas [6]. Neuwirth in 1976 described the first “excision of submucous fibroids with hysteroscopic control,” performed by classical slicing technique. Although this new surgical approach was a breakthrough in the treatment of submucous myomas, the authors recommended resectoscopic myomectomy be performed only by expert endoscopic surgeons [7, 8]. Indeed, the main limit in performing hysteroscopic myomectomy has always been represented by the intramural component of submucous myomas, as it is responsible for unsatisfactory surgical outcomes, intraoperative complications, and need for repeated procedures [9, 10]. The difficulty to manage submucous myomas with a deep myometrial development was well described and demonstrated by Wamsteker in 1993, conceiving a new classification—still used today—based on the amount of intramural component of submucous myomas. The authors suggested limiting the treatment of deeper submucous myomas only in selected cases because it correlated with high risk of repeated procedures [9].

Over the last decades, there has been a rapid evolution in the hysteroscopic approach for the treatment of submucous myomas, driven by the technological progress that has offered a wide range of performing instruments to the surgeons. At the same time, several techniques for in-patient hysteroscopic myomectomy have been proposed aimed at ensuring the safe and effective removal of submucous myomas. Among them, in order to minimize the need of repeated procedures, the authors conceived techniques to accomplish the treatment in only one surgical time [11], as multiple treatments can cause physical and mental stress for both surgeons and patients, along with a higher surgical risk [12]. To date, a comprehensive analysis on the success rate of in-patient hysteroscopic myomectomy in a single treatment, according to the technique applied, is lacking in scientific literature.

The aim of this systematic review and meta-analysis was to assess the feasibility of the one-step in-patient hysteroscopic myomectomy according to the technique adopted. Moreover, as a secondary outcome, the rate of intraoperative complications recorded in the selected clinical series, when reported, was also meta-analysed to assess the safety of each technique.

## 2. Methods

### 2.1. Protocol

The review was reported following the PRISMA guidelines for reporting systematic reviews and meta-analyses [13] and registered in the PROSPERO database (registration number: CRD42017067543).

Prospective or retrospective clinical series, cohorts and case control studies, and randomized controlled trials were considered eligible for the review. Medical papers reporting feasibility of the one-step procedure, the technique by which the in-patient hysteroscopic myomectomies were performed, as well as the characteristics of patients and myomas treated were considered for the meta-analysis. If available, additional information such as myoma size, number/rate of cases with multiple myomas in the series, administration of gonadotropin releasing hormone analogue (GnRHa), or other presurgical therapies and intraoperative complications were also recorded. In the absence of information, the corresponding authors, if available, were contacted to provide them.

### 2.2. Literature Search and Review

A scientific literature search was performed in July 2016 and was conducted in main databases including PubMed, ClinicalTrials.gov (http://www.clinicaltrials.gov), SCOPUS, Scielo, and AJOL (African Journals Online) search engines, using combinations of the following keywords: “operative hysteroscopy” AND “myomectomy,” “operative hysteroscopy” AND “complications,” “hysteroscopic myomectomy,” “hysteroscopic complication” AND “myomectomy.” No language limits were set. In order to better categorise and analyse the one-step feasibility rate according to the kind of myomas treated, only medical papers reporting submucous myomas categorized according to the Wamsteker Classification (G0, completely intracavitary, pedunculated myoma, with no intramural extension; G1, submucous myoma with <50% intramural extension; and G2, submucous myoma with at least 50% intramural extension) [9] were considered as eligible. Therefore, clinical series published before 1993 were excluded from the review.

Medical papers were assessed in a multistep procedure as follows. Titles and abstracts were evaluated, and duplicates were discarded. Case reports, reviews, overviews, letters, guidelines, meta-analyses, and surveys with questionnaires were not considered eligible for meta-analysis. Studies about in-office hysteroscopic myomectomy were also excluded due to the different settings which could bias the comparison with in-patient procedures. Additional articles incidentally found during the full-text research and references discovered by reading the selected medical papers were also introduced in the meta-analysis database. English, French, Spanish, Portuguese, and Italian full-text articles were read in original languages. Other medical papers were translated in English or Italian.

### 2.3. Data Extraction

Feasibility rate of one-step in-patient hysteroscopic myomectomy was the main effect size assessed and was defined as the rate of cases in which in-patient hysteroscopic myomectomy was accomplished in only one surgical procedure, entirely removing submucous myomas treated. As a secondary effect size, the rate of complications found during the hysteroscopic procedure or immediately after the surgery was calculated. Long-term complications were not considered.

Rates were calculated per protocol and the rate of the event was assessed as a binomial casual variable. Two authors (I. U. and F. A.) performed data extraction at the same time by reading the texts altogether. Any disagreement between them over the eligibility of specific studies was resolved through discussion with a third author (B. V.). In case of complications or feasibility rates of 0% or 100%, the rate of rare events was calculated applying the rule of Quigley et al. (i.e., 2/5*n* in case 0 events occur in order to estimate rare events, where *n* is the number of observations in the sample) [14].

Missing information was asked to corresponding authors by e-mail or phone call.

### 2.4. Quality Assessment

The quality assessment of the series was evaluated based on the feasibility reported in accomplishing the in-patient hysteroscopic myomectomy in only one-step procedure. Two authors (F. A. and I. U.) provided the quality assessment altogether, and disagreements were resolved by discussion with a third author (B. V.). A quality score system in accordance with the GRADE approach [15] was used for assessing the quality of each series included in meta-analysis. Considering that studied effect size was expressed as a rate, the GRADE score was modified assuming additional sources of bias.

Clinical series were scored as follows:

- 4 for randomized controlled trial
- 3 for prospective studies
- 2 for retrospective studies
- 1 for small series

Complications in operative hysteroscopy are rare [16–18] but when trying to avoid them, they can influence the one-step procedure feasibility [19]. Some small series could not report complications, while other small series could report complications, leading to underestimation or overestimation of the complications rate. Therefore, during the selection of the clinical series, it was considered that the minimal limit of cases for including a small series was the one with at least a complication. Nevertheless, if other small series with the same number of patients enrolled were found but with no complications reported, they also were included in meta-analysis. This choice was given to balance the overestimation or underestimation of complications rates in small series. Small series were considered if characterised by a total of 14 cases or less.

The quality assessment downgraded the small series even when they were a part of other kind of studies (i.e., arms of randomized controlled trials or observational studies).

Additional sources of biases were considered, and the score was downgraded or upgraded as follows:

- Surgical technique description was not clearly available (−1: poorly explained, +1 clearly explained).
- Feasibility was not clearly reported (−1: poorly reported, +1 clearly reported).
- The characteristics of patients were not clearly described (−1: poorly described, +1 clearly described).
- Need of estimating missing data (−1: yes; +1: no) in the feasibility if the reported rate of success in one-step myomectomy was 100% or 0% (need to estimate rare events) or missing mean myoma size as mean diameter.
- Sample of ≤100 cases (−1: yes; +1: no).

To get the overall quality score, this bias score was added to the one given according to the type of study as previously described. A score of more than 4 was considered to assess good quality series.

### 2.5. Statistical Analysis

Data were extracted from selected studies and combined applying a random-effect model [20], which incorporates heterogeneity of effects. Heterogeneity of studies was evaluated by the Cochrane *Q* test and reported as *I*^2^ statistics, which describe the percentage of total variation across studies which is due to heterogeneity rather than chance [21]. Heterogeneity was considered significant if *p* < 0.10 and *I*^2^ was more than 50%. Begg and Egger's tests [22, 23] were used to test for publication bias. In a sensitivity analysis, the influence of individual studies on pooled estimates was assessed using Tobias' method [24]. If the point estimate with one study omitted lay outside the confidence interval (CI) of the overall estimate of all trials, the study was indicated as having excessive influence.

### 2.6. Subgroup Analyses

On the basis of the main aim of this meta-analysis and on what already reported by Wamsteker et al. about difficulties of treating the intramural component of submucous myomas [9], the series were arranged in subgroups taking into consideration the technique for removing the myomas and the proportion of the G2 myomas reported in the series (more or equal to 50% or less than 50%).

The z-statistic (one-tiled) was applied to compare the subgroups effect sizes (mean feasibility and mean rate of complications) in subgroups. A *p* < 0.05 was set as significant.

The calculations were made by using StatsDirect Software, version 2.7.2 (Cheshire, UK, 2008).

## 3. Results

**Figure 1:**
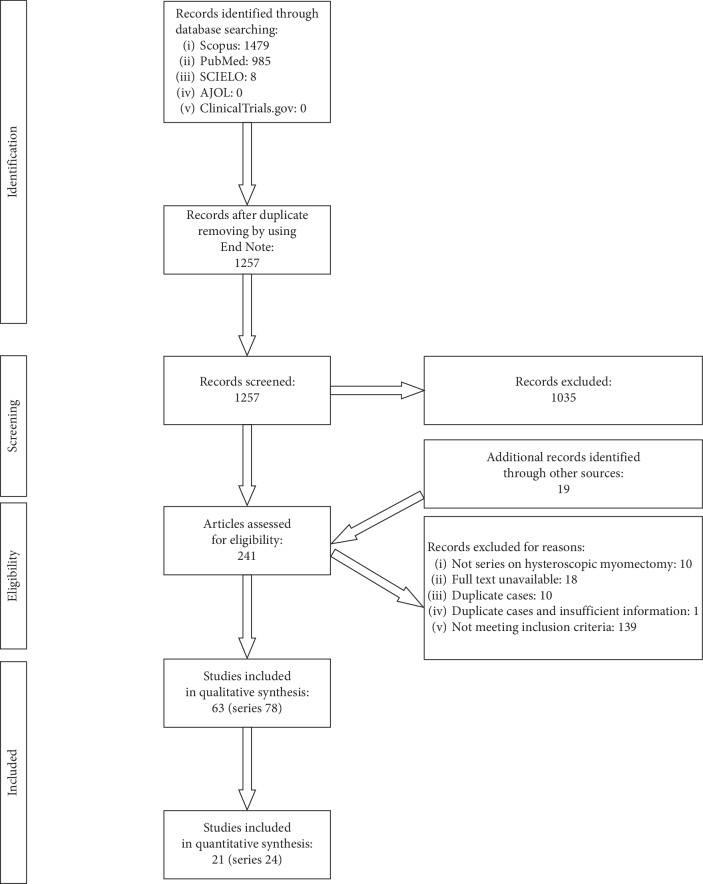
Flow chart of the phases for selecting studies and series.

The steps of study selection are reported in Figure 1 (PRISMA flow diagram). During the literature review, 2472 references were found. There were 1215 duplicate references, which were removed by using the EndNote tool. After removing duplicates, 1257 references were screened by reading the titles and abstracts, looking for clinical series of in-patient operative hysteroscopies. Additional duplicate references were removed manually. Five hundred and ninety-four studies were reassessed focusing on operative hysteroscopies, excluding out-patient procedures. Two hundred and seventy-nine studies were reassessed looking for hysteroscopic myomectomies even in subgroups. In this step, the surveys based on questionnaires were also excluded. Twelve studies were removed from the database because they were published before 1993. Full texts of 222 studies were searched. Looking for full-texts, 19 more studies were found and were added to the database. The studies in the database were carefully checked for eligibility. Ten studies were discarded because after the full-text reading, it was understood that they were not clinical series on hysteroscopic myomectomy, while 18 other studies were discarded because the full texts were not available. Of the 213 studies, 11 were discarded because 10 of them reported duplicate cases and 1 study reported a series with duplicate cases in one arm and insufficient information in the other arm. One hundred and thirty-nine studies were discarded because they did not meet the inclusion criteria. Sixty-three references were eligible for the review [10, 12, 17, 25–50, 51–84] (Table 1). Thirteen studies reported two or more arms. Each arm was able to be meta-analysed for feasibility. Therefore, 78 effect sizes were assessed. Instead, 73 series were adequate to be assessed for complications rate [10, 12, 17, 25–29, 31–39, 41–76, 79–84].

The 78 series came from 20 different countries: 26 from Italy (33.3%), followed by France (9 series, 11.5%), United States, China, Spain (5 series, 6.4%), Egypt (4 series, 5.1%), United Kingdom, Turkey, Japan (3 series, 3.8%), Tunisia, Poland, Greece, the Netherlands (2 series, 2.6%), Australia, Belgium, Brazil, Germany, Taiwan, Finland, and India (1 series, 1.3%).

Series of poor quality (≤4 in quality score) were 54 (69.2%). The characteristics of the selected study and the quality score given for each series are reported in Table 1.

With regard to the techniques applied, classical slicing was the most commonly used technique for removing submucous myomas (45 series, 57.7%). In 6 series (7.7%), morcellators (Truclear®, MyoSure®, and Bigatti shaver®) were used. In one series (1.3%), the YAG laser was used (Table 1). In the series of Smets et al. [77], morcellators, YAG laser, and classical slicing were all used.

The hysteroscopic myomectomy techniques conceived for treating the intramural component of fibroid in only one surgical step are summarized in Table 2.

**Figure 2:**
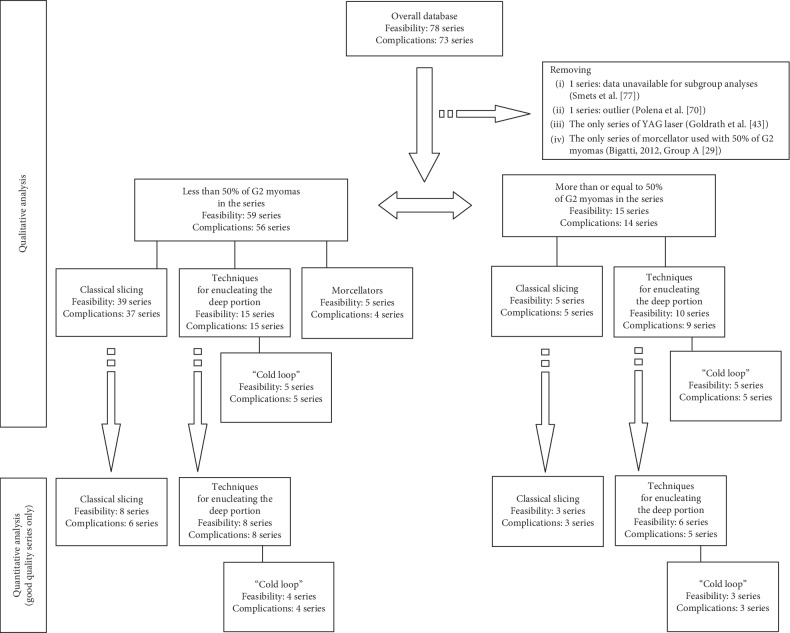
Flow chart of the organization of subgroups.

Figure 2 shows in details the subgroup arrangement for qualitative and quantitative analysis. The good quality series (>4 quality score) were 25. In total, 3037 and 2888 patients were considered to study feasibility and complications rates, respectively. Of the 25 series of good quality, 10 used the slicing technique (7 with <50% of G2 myomas rate, while 3 with ≥50% of G2 myomas rate). One series reported the use of Myosure® [46]. Techniques for enucleating the deep portion of myomas were reported in 14 series (8 with <50% of G2 myomas rate, while 6 with ≥50% of G2 myomas rate). The “Cold loop” technique [17] was reported in 7 series (4 with <50% of G2 myomas rate and 3 with ≥50% of G2 myomas rate) (Table 1). Complications were not assessed in 2 [40, 78] of 25 series.  Table 3 reports the quality score results in assessing bias risk, of the 24 series of good quality available for meta-analysis. The Myosure® arm series in Hamidouche et al. [46] is not reported in Table 3 because it was the only good quality series of the morcellators group. Therefore, it was not possible to meta-analyse data on the morcellators group.

**Figure 3:**
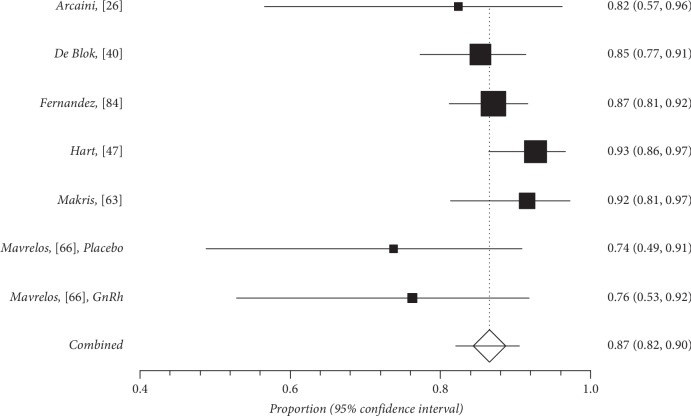
Forest plot of slicing technique feasibility in series with less than 50% of G2 myoma rate.

**Figure 4:**
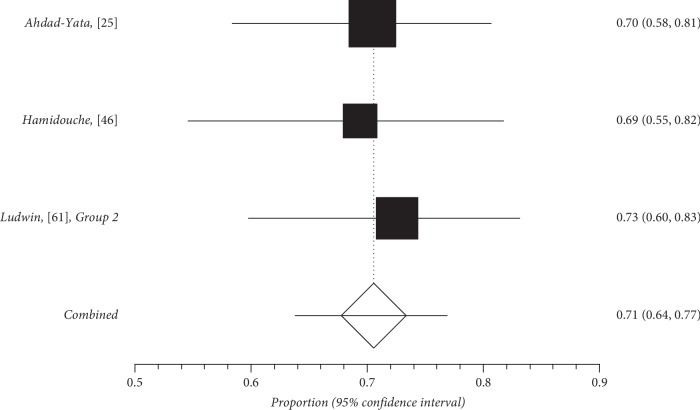
Forest plot of slicing technique feasibility in series with at least 50% of G2 myoma rate.

**Figure 5:**
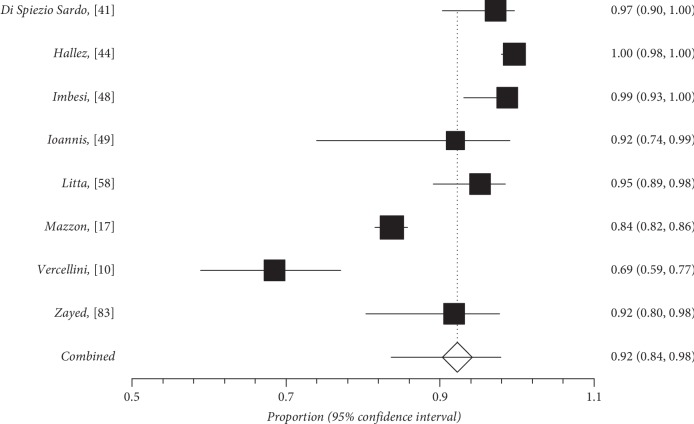
Forest plot of the feasibility of techniques conceived to enucleate the deep portion of myomas in series with less than 50% of G2 myoma rate.

**Figure 6:**
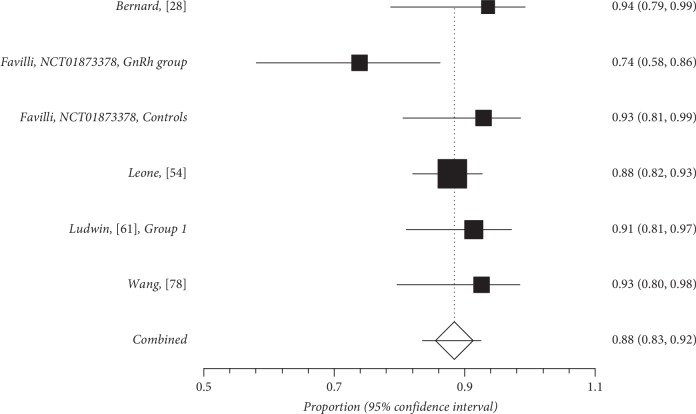
Forest plot of the feasibility of techniques conceived to enucleate the deep portion of myomas in series with at least 50% of G2 myoma rate.

**Figure 7:**
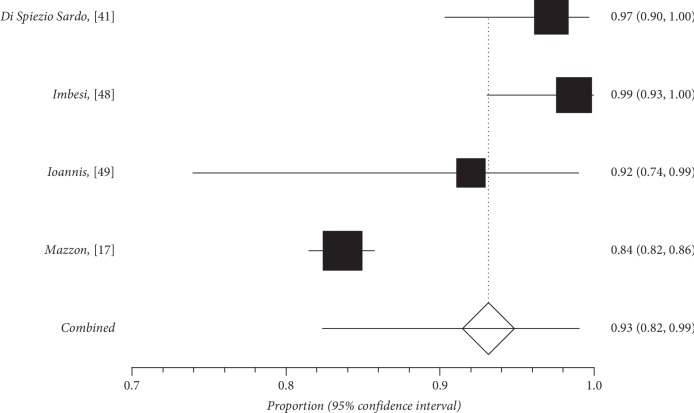
Forest plot of “Cold loop” feasibility in series with less than 50% of G2 myoma rate.

**Figure 8:**
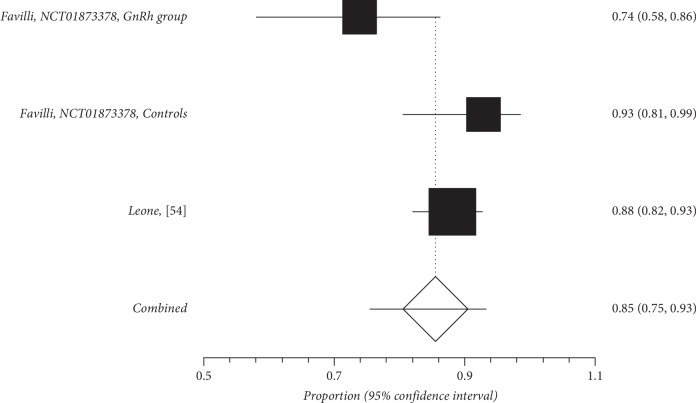
Forest plot of “Cold loop” feasibility in series with at least 50% of G2 myoma rate.

Quantitative subgroup findings were reported in Table 4. The forest plots for the feasibility proportions are shown in Figures 3to 8. Figures 3 and 4 report forest plots for the slicing technique. Figures 5 and 6 report forest plots for the techniques used for removing the deep portion of myomas. Figures 7 and 8 report forest plots for the “Cold loop” technique. The figures report data syntheses of series with less than 50% rates of G2 myomas and at least 50% rate of G2 myomas.

The Myosure® arm series in Hamidouche et al. study [46] (the only good quality series among morcellators group) reports 0.647 (95% CI 0.476–0.787) of feasibility, while complications cases were 5 to 34 patients (0.147; 95% CI 0.063–0.308). All complications reported in the study of Hamidouche at al [46] were instances of bleeding.

Table 5 reports descriptive statistics of complications rates found in good quality studies.

The comparison among groups of good quality series resulted in the following:

- Slicing technique versus techniques for enucleating deep portion of myomas in clinical series with less than 50% of G2 myomas. Feasibility: *z* = 5.454, *p* < 0.001. Complication rate: *z* = 6.277, *p* < 0.001.
- Slicing technique versus “Cold loop” technique in clinical series with less than 50% of G2 myomas. Feasibility: *z* = 6.303, *p* < 0.001. Complication rate: *z* = 4.044, *p* < 0.001.
- Slicing technique versus techniques for enucleating deep portion of myomas in clinical series with more or equal to 50% of G2 myomas. Feasibility: *z* = 5.000, *p* < 0.001. Complication rate: *z* = 1.846, *p* < 0.066.
- Slicing technique versus “Cold loop” technique in clinical series with more or equal to 50% of G2 myomas. Feasibility: *z* = 3.608, *p* < 0.001. Complication rate: *z* = 3.712, *p* < 0.002.

## 4. Discussion

The main objective of the present systematic review and meta-analysis was to assess the feasibility rate of the one-step in-patient hysteroscopic myomectomy according to the technique adopted. Therefore, the results obtained from this meta-analysis should be considered with a descriptive value. To our knowledge, this is the first meta-analysis on this subject.

The high heterogeneity observed in accomplishing in-patient hysteroscopic myomectomies in only one-step procedures, even after sub-groups analysis, was the main finding that emerged from the review of current literature. Therefore, it is difficult to provide the true rate of feasibility and complications according to techniques applied, based on the available literature. Indeed, a significant number of poor quality series—due to bias on collecting or reporting data—were found in the scientific literature available. Although myoma grading is reported in clinical series published after the advent of Wamsteker classification [9], the number of submucous myomas was missing at times and in the same way, the location of myomas was often not reported. The mean myoma size was also often not described in detail. Some authors reported the main diameter of myomas as mean or median with interquartile ranges, suggesting asymmetric distribution of myoma size. Additionally, in some cases, the authors provided the myomas' size as mean diameter using ultrasound investigation (trans-vaginal or trans-abdominal scans); in other ones, they used radiological imaging techniques or subjective assessment during hysteroscopy. Moreover, all these methods might be imprecise in assessing myomas' size, due to the irregular shape of fibroids. It should be underlined that with an increasing diameter, the volume of myoma grows to the third power. This issue greatly affects the complete removal of myoma in one-step surgical procedures [85].

Doubtless, the intramural extension of submucous fibroids influences the chance of achieving the complete resection of myomas in one surgical session [11]. As acknowledged by the authors [86, 87], the possibility to perform an in-patient hysteroscopic myomectomy with a low complications rate is also linked to several parameters related to the myomas (volume, number, grading, and location). Even in good quality series, all those factors related to the myoma characteristics may play a role in influencing the rate of incomplete removal of myomas, justifying the heterogeneity found.

The wide variability highlighted seems also to reflect the personal ability of surgeons to deal with submucous myomas, according to their skills and surgical background, as several techniques for removing deep submucous myomas have been reported (Table 2).

An additional source of variability may be the use of drugs before the hysteroscopic myomectomy or other concomitant surgeries in the same procedure. It is currently unclear if other surgeries or therapies could affect the one-step hysteroscopic myomectomy [42].

The use of alternative techniques for removing the intramural portion of the myomas seems to improve feasibility compared to the slicing technique, with less or equal rate of intraoperative complications. Nevertheless, none of the techniques for treating the deep portion of myomas have been tested against the slicing technique or against other techniques in randomized controlled trials. Therefore, it is not possible to label a single technique as the best one. Among techniques for treating the intramural portion of the myomas, only the “Cold loop” technique has been reported by different authors, demonstrating a certain degree of reproducibility.

The use of morcellators seems to be limited to series with a low rate of or with no G2 myomas. In the present systematic review, only one good quality series describing morcellators was available for meta-analysis, it was therefore not possible to carry out data synthesis.

Finally, caution in interpreting the rate of complications should be used. It was decided to provide results of complications as a secondary outcome because the effectiveness of a surgical technique cannot be assessed without taking into consideration intraoperative complications. As the quality assessment was only done on the feasibility rate, bias on the reports of complications could be found even in good quality studies. However, it has already been acknowledged that the complications of operative hysteroscopy are overall low [16–18], in agreement with the findings of the present study.

Based on the findings of this meta-analysis, it can be stated that it is hard to compare the feasibility and the complications rates of the resectoscopic myomectomy according to the technique adopted among available clinical series. The studies often do not report pivotal information to allow comparability. Future clinical series on in-patient hysteroscopic myomectomy should provide a detailed description of the myomas treated and of the characteristics of patients treated, along with information on additional hysteroscopic procedures needed to accomplish the treatment and any presurgical therapy administered.

## 5. Conclusions

In conclusion, it can be stated that there is still no single hysteroscopic technique proven to be unequivocally superior to the others for treating submucous fibroids with intramural development in one-surgical step. Nevertheless, despite the heterogeneity found among the clinical series analysed, it seems that all the techniques used to deal with the intramural portion of myomas work better than the slicing technique, achieving a higher rate of procedures accomplished in a single surgical time and a lower number of complications. Randomized controlled trials for testing which is the best technique for the one-step in-patient hysteroscopic myomectomy are needed. In absence of such evidence, it should be assumed that classical slicing is not the best surgical technique for treating the intramural portion of the myomas.

## Figures and Tables

**Table 1:** Description of series in each study.

Author	Country	Type of study quality score	Patients treated	Mean age	Other surgeries	Rate of GnRh agonist	Technique	Some multiple myomas	Rate of G2	Mean myoma size reported as main diameter (cm)	Notes
Ahdad-Yata 2015 [25]	France	Retrospective 5	71	38.4	No	7.0%	Classical slicing	Yes	59.0%	2.8	

Arcaini 1994 [26]	Italy	Prospective 6	17	43.4	No	100%	Classical slicing	Yes	0	1.4	Provided missing information

Arnold 2016 [27]	Australia	Prospective 2	95	47.5	No	0	MyoSure®	Yes	17.9%	4.0	

Bernard 2000 [28]	France	Retrospective 5	31	35	Yes	0	Hydromassage	Yes	51.6%	2.0	Provided missing information

Bigatti 2012 [29]	Italy	Randomized									
Group A											
		2	12	49.4	No	0	Bigatti Shaver®	No	50%	2.0	Provided missing information
Group B		0	3	47.7	No	0	Classical slicing	No	0	1.8	

Bigatti 2014 [30]	Italy	Retrospective									
Group A											
Group B		3	76	47.6	Missing	0	Bigatti Shaver®	Yes	36.4%	2.2	
		3	51	48	Missing	0	Classical slicing	Yes	35.8%	2.5	

Bizzarri 2015 [31]	Italy	Prospective									
Group S		2	23	35	No	0	Classical slicing	No	38.7%	Missing	S: direct surgery
Group T		2	20	36.3	No	100%	Classical slicing	No	42.3%	Missing	T: triptorelin
Group L		0	11	36.8	No	0	Classical slicing	No	50.0%	Missing	L: letrozole
Group U		0	7	38.4	No	0	Classical slicing	No	40.0%	Missing	U: ulipristal

Blanc 1997 [32]	Francex	Prospective 4	196	41	Yes	18.9%	Classical slicing	Yes	4.1%	2.3	

Bori 2011 [33]	Italy	Retrospective 3	80	41	Yes	1.0%	“Cold loop”	Yes	13.0%	3.0	Provided missing information

Bourdel 2010 [34]	France	Retrospective 3	72	45.6	Missing	0	Classical slicing	Yes	12.5%	2.3	

Camanni 2010 [35]	Italy	Prospective 4	33	41.3	Yes	9.1%	“Cold loop”	Yes	60.2%	5.5	Provided missing information

Campo 2005 [36]	Italy	Prospective									
Group A		2	42	38.8	Yes	0%	Classical slicing	Yes	21.4%	2.9	
Group B		2	38	39	Yes		Classical slicing	Yes	18.4%	3.0	

Casadio 2011 [37]	Italy	Prospective 0	13	36.5	No	0	“Cold loop”	No	100%	Missing	

Chelli 2006 [38]	Tunisia	Retrospective 3	125	40.2	Yes	Missing	Classical slicing	Yes	22.0%	2.9	

Darwish 2003 [39]	Egypt	Prospective									
Group A		2	65	21.7	No	0	Technique for deep portion	No	0	Missing	
Group B		2	77	25.1	No	0	Classical slicing	No	0	Missing	

De Blok 1995 [40]	The Netherlands	Retrospective 5	109	40	Missing	100%	Classical slicing	Missing	11.0%	1.1	

Di Spiezio Sardo 2015 [41]	Italy	Prospective 6	72	38	Yes	69.4%	“Cold loop”	Yes	33.3%	4.1	

Favilli [42] NCT01873378	Italy	Randomized									Raw data available
GnRH group		7	42	40.5	No	100%	“Cold loop”	No	57.1%	2.8	
Controls		7	42	40.9	No	0	“Cold loop”	No	57.1%	2.5	

Fernandez 2001 [84]	France	Retrospective 5	177	42.2	Missing	13%	Classical slicing	Yes	17.0%	2.9	

Goldrath 1997 [43]	USA	Small series 2	3	31	Yes	0	YAG laser destruction	Yes	66.7%	Missing	

Hallez 1995 [44]	France	Retrospective 6	274	45	Yes	Missing	Manual massage	Yes	34.7%	Missing	

Hamerlynck 2011 [45]	The Netherlands	Retrospective 1	37	41.3	Missing	0	Truclear®	Missing	8.1%	2.0	

Hamidouche 2015 [46]	France	Retrospective								G0, G1, G2 mean diameters in both arms	
MyoSure		5	34	40.8	Yes	0	MyoSure®	Yes	41.7%		
Bipolar use		5	49	40.2	Yes	0	Classical slicing	Yes	63.3%		

Hart 1996 [47]	United Kingdom	Prospective 6	122	43.2	Yes	Missing	Classical slicing	Yes	33.0%	3.4	

Imbesi 2008 [48]	Italy	Prospective 6	78	43.9	Yes	65.4%	“Cold loop”	Yes	26.9%	2.8	

Ioannis 2006 [49]	Greece	Prospective 6	25	30.1	Missing	24.0%	“Cold loop”	No	16.0%	2.3	

Jayakrishnan 2013 [50]	India	Prospective 1	37	32	Yes	5.4%	Classical slicing and laparoscopy	Missing	5.4%	Missing	

Kim 1995 [51]	USA	Small series -2	6	42	No	33.3%	Classical slicing	Yes	16.7%	Missing	

Korkmazer 2016 [52]	Turkey	Prospective 4	64	43.9	No	0	Technique for deep portion	No	28.1%	4.0	

Lasmar 2004 [53]	Brazil	Retrospective 3	44	43.5	Missing	0	Technique for deep portion	Yes	34.1%	2.2	Provided missing information

Leone 2012 [54]	Italy	Prospective 6	159	44	Yes	50.9%	“Cold loop”	Yes	54.1%	Missing	

Lin 2012 [55]	Japan	Prospective 2	1569	38.6	Missing	Not reported	Technique for deep portion	Yes	1.2%	Missing	

Lin 1994 [56]	Japan	Prospective 4	25	38	No	100%	Technique for deep portion	No	16.0%	Missing	

Litta 2014 [58]	Italy	Retrospective 7	104	35.7	No	19.2%	Technique for deep portion	Yes	32.7%	2.8	

Litta 2003 [57]	Italy	Prospective 4	41	42.1	No	82.9%	Technique for deep portion	No	100%	3.2	

Loffer I 2005 [59]	USA	Retrospective 1	20	63.3	Yes	0	Classical slicing Sometimes scissors	Yes	15.0%	2.4	

Loffer II 2005 [60]	USA	Retrospective									EA: endometrial ablation
With EA		1	73	44	Yes	79.4%	Classical slicing	Yes	20.5%	3.0	
Without EA		3	104	37.6	No	21.2%	Classical slicing	Yes	29.8%	3.4	

Ludwin 2013 [61]	Poland	Prospective									
Group 1		6	58	37.2	Yes	17.2%	Technique for deep portion	No	63.8%	2.4	
Group 2		6	62	37.3	Yes	21.0%	Classical slicing	No	50.0%	2.4	

Lure 1999 [62]	Spain	Retrospective 1	143	45	Yes	62.3%	Classical slicing	Yes	16.9%	Missing	

Makris 2007 [63]	Greece	Prospective 6	59	34.6	Missing	100%	Classical slicing	No	16.9%	1.5	

Malek-Mellouli 2012 [64]	Tunisia	Retrospective 1	105	41.4	Missing	Missing	Classical slicing	Yes	5.8%	3.1	

Marziani 2005 [65]	Italy	Prospective 4	107	38	Missing	Not reported	Classical slicing	Yes	12.1%	Missing	Provided missing information

Mavrelos 2014 [66]	United Kingdom	Randomized									Provided missing information
Placebo		5	19	44.5	Missing	0	Classical slicing	Yes	0	2.9	
GnRh arm		5	21	38.8	Missing	100%	Classical slicing	Yes	0	3.1	

Mazzon 2015 [17]	Italy	Retrospective 5	1215	42	Yes	60.4%	“Cold loop”	Yes	49.8%	2.0	

Muñoz 2003 [67]	Spain	Retrospective 1	120	44.8	Yes	60.0%	Classical slicing	Yes	14.2%	Missing	

Murakami 2008 [12]	Japan	Prospective 2	28	35.1	No	Missing	Technique for deep portion	Yes	78.6%	Missing	Provided missing information

Muzii 2010 [68]	Italy	Randomized									
Group A		3	20	42	No	100%	Classical slicing	Yes	0	1.9	
Group B		3	19	42	No	0	Classical slicing	Yes	0	2.0	

Namazov 2015 [69]	Turkey	Retrospective 1	98	35	Missing	0	Classical slicing	No	4.1%	Missing	

Polena 2007 [70]	France	Retrospective 3	235	47.9	Yes	3%	Classical slicing	Yes	70.0%	Missing	

Romer 1997 [71]	Germany	Prospective 4	70	41	Missing	28.6%	Classical slicing	No	34.3%	Missing	

Rovio 2009 [72]	Finland	Prospective 4	53	44.7	Yes	0	Classical slicing	Yes	0	2.1	

Rovira Pampalona 2012 [73]	Spain	Retrospective 1	76	47	Yes	0	Truclear®	No	0	Missing	

Sancho 2016 [74]	Spain	Retrospective									
Ulipristal		1	26	44	Missing	0	Classical slicing	Yes	50.0%	3.4	
GnRh agonist		1	24	38	Missing	100%	Classical slicing	Yes	36.0%	3.4	

Şendağ 2013 [75]	Turkey	Retrospective 3	40	35	Missing	Missing	Classical slicing	Yes	20.0%	2.2	

Shokeir 2005 [76]	Egypt	Prospective 4	29	31.4	Missing	Missing	Classical slicing	No	0	1.3	

Smets 1996 [77]	Belgium	Retrospective 1	366	33	Yes	100%	Classical slicing, morcellator, or YAG laser	Yes	23.8	Missing	

Vercellini 1999 [10]	Italy	Prospective 7	108	37.3	Yes	82.4%	Technique for deep portion	Yes	22.2%	G0, G1, G2 mean diameters	

Wang 2016 [78]	China	Retrospective 5	40	32.6	No	0	Technique for deep portion	No	100%	4.0	

Wong 2013 [79]	China	Small series 2	5	41.6	Missing	0	Classical slicing	Yes	20.0%	2.9	

Wong 2014 [80]	China	Randomized									
Vasopressin arm		1	20	41.6	Missing	0	Classical slicing	Yes	15.0%	Missing	
Placebo		1	19	42.9	Missing	0	Classical slicing	Yes	10.5%	Missing	

Xia [81] 2005	China	Retrospective 3	877	44	Yes	32.2%	Classical slicing	Yes	32.2%	3.9	

Yen [82] 2007	Taiwan	Small series 2	5	28	No	20.0%	Classical slicing	Yes	40.0%	2.8	

Zayed [83] 2015	Egypt	Prospective 6	49	37.6	Missing	0	Technique for deep portion	Yes	46.9%	6.0	

Studies are listed alphabetically on the first left column. The characteristics of the series are reported along with quality score given. The description of the techniques for treating the deep portion of the myoma is wider reported in Table 2.

**Table 2:** Summary of techniques reported for removing the deep portion of myomas.

Author	Short description of the technique for treating the deep portion
Bernard [28]	Inducing uterine contraction by changing intrauterine pressure (“hydromassage”)

Darwish [39] Group A.	Vertical linear incision of the myoma to facilitate the sliding into the endometrial cavity. Ergometrine administration to promote uterine contractions. The base was cut and the whole myoma extracted through the primed cervical canal using a ring forceps.

Hallez [44]	Massage of the uterus manually, applying a pressure on the deep portion of myoma (so-called “manual massage”)

Jayakrishnan, 2013 [50]	Classical slicing under laparoscopic check in 86.5% of patients. Laparoscopic removal of larger myomas with intramural portion

Korkmazer [52]	Cavitation of the cleavage. The cleavage was detected by transabdominal ultrasonography. Then, slicing of the deep portion under transabdominal sonographic check.

Lasmar [53]	Collins' electrode was used to encircle the entire myoma and to reach the pseudocapsule. From this point, the fibroid was mobilized and the fibrous bundles were individualized and sectioned with electrical energy.

Lin [56]	Cutting the pseudocapsule of the myoma. Lin' grasper for pulling the deep portion into uterine cavity. Slicing under ultrasonographic check.

Litta [55]	Elliptical incision of the mucosa that covers the myoma at the level of uterine wall and detection of the cleavage. Cutting of the fibrous bridges between myoma and uterine wall, thereby obtaining expulsion of the deep portion into uterine cavity.

Ludwin [61], Group I.	Classic slicing and cut of pseudocapsule, under trans rectal ultrasonographic check.

Murakami [12]	Resection of the intrauterine dome of the myoma. Induction of strong contraction by using PGF2alpha within uterine body. Slicing or vaporization of the deep portion. Sometimes, mechanical detachment. Echographic check.

Vercellini [10]	Deactivated electrode within the cleavage for pulling and detaching the deep portion from the uterine wall.

Wang [78]	Exposing the myoma edges by cutting endometrium close to the myoma dome. Classic slicing. Oxytocin for inducing contractions in case of large myomas and forceps for pulling the residual portion of the deep myoma. Echographic check.

Zayed [83]	Introducing the loop into the cleavage; traction of the deep portion into uterine cavity. Hydromassage. Manual massage. Echographic check. Multiple slicing session after each induced protrusion of the myoma into uterine cavity.

Mazzon [17]	“Cold loop”: classic slicing of the intrauterine portion of the myoma. Exposure of the pseudocapsule. Change of the loop and use of the cold loop to mobilize the myoma from the uterine wall thereby pulling the deep portion into uterine cavity.

The description of the techniques for treating the deep portion of the myomas is usually reported in the texts. Sometimes, the authors recall the papers where the techniques have been described.

**Table 3:** Quality score results.

	Series	Modified GRADE score	Wide explanations on surgical techniques	Clearly reporting on feasibility	Characteristics of patients disclosed	Missing myoma' diameter as mean	Sample size	Total
Classical slicing in series with less than 50% of G2	Arcaini [26]	3	1	1	1	1	−1	6
	De Blok [40]	2	−1	1	1	1	1	5
	Fernandez [84]	2	1	−1	1	1	1	5
	Hart [47]	3	−1	1	1	1	1	6
	Makris [63]	3	1	1	1	1	−1	6
	Mavrelos, 2010, Placebo [66]	4	−1	1	1	1	−1	5
	Mavrelos, GnRh [66]	4	−1	1	1	1	−1	5

Techniques for treating the deep portion (including “cold loop”) of the myomas in series with less than 50% of G2	Di Spiezio Srado [41]	3	1	1	1	1	−1	6
	Hallez [44]	2	1	1	1	−1	1	6
	Imbesi [48]	3	1	1	1	1	−1	6
	Ioannis [49]	3	1	1	1	1	−1	6
	Litta [58]	2	1	1	1	1	1	7
	Mazzon [17]	2	1	1	−1	1	1	5
	Vercellini [10]	3	1	1	1	1	1	7
	Zayed [83]	3	1	1	1	1	−1	6

Classical slicing in series with more or equal to 50% of G2	Ahdad-Yata [25]	2	1	1	1	1	−1	5
	Hamidouche [46]	2	1	1	1	1	−1	5
	Ludwin, Group 2 [61]	3	1	1	1	1	−1	6

Techniques for treating the deep portion of the myomas (including “cold loop”) in series with more than or equal to 50% of G2	Bernard [28]	2	1	1	1	1	−1	5
	Favilli, GnRh group [42]	4	1	1	1	1	−1	7
	Favilli, Controls [42]	4	1	1	1	1	−1	7
	Leone [54]	3	1	1	1	−1	1	6
	Ludwin, Group 1 [61]	3	1	1	1	1	−1	6
	Wang [78]	2	1	1	1	1	−1	5

Quality score results for studies judged of good quality (quality score more than 4). None of the good quality series falls among the ones in which estimating the rare event has been needed. In the study of Leone et al. [54], the main myoma diameter was reported as median. Hallez et al. [44] provided intervals for diameters of myoma.

**Table 4:** Results of data syntheses.

	Less than 50% of G2 myoma rate in clinical series	At least of 50% of G2 myoma rate in clinical series
*Slicing technique in clinical series*		
Feasibility	0.865	0.706
	95% CI: 0.820–0.904	95% CI: 0.638–0.769
	*I* ^2^: 41.8%, *p*=0.112	*I* ^2^: 0%, *p*=0.928
	Begg's risk of bias: −0.524, *p*=0.069	Begg and Egger's risk of bias cannot be calculated (too few strata)
	Egger's risk of bias: −1.583, *p*=0.083	
Complication rate	0.0560	0.0686
	95% CI: 0.0301–0.0894	95% CI: 0.0092–0.1766
	*I* ^2^: 31.8%, *p*=0.197	*I* ^2^: 81.6%, *p*=0.004
	Begg's risk of bias: 0.467, *p*=0.272;	Begg's and Egger's risk of bias cannot be calculated (too few strata)
	Egger's risk of bias 0.972, *p*=0.336.	

*Techniques for enucleating the deep portion of myomas*		
Feasibility	0.923	0.882
	95% CI: 0.836–0.978	95% CI: 0.835–0.925
	*I* ^2^: 96.1%, *p* < 0.001	*I* ^2^: 41.6%, *p*=0.128
	Begg's risk of bias: −0.333, *p*=0.239	Begg's risk of bias: 0.2, *p*=0.719
	Egger's risk of bias −3.913, *p*=0.279	Egger's risk of bias −0.627, *p*=0.743
Complication rate	0.0102	0.0393
	95% CI: 0.0062–0.0152	95% CI: 0.0204–0.0640
	*I* ^2^: 0%, *p*=0.569	*I* ^2^: 0%, *p*=0.510
	Begg's risk of bias 0.357, *p*=0.275	Begg's risk of bias: 0.571, *p*=0.173
	Egger's risk of bias 0.169, *p*=0.627	Egger's risk of bias: 0.787, *p*=0.408

*“Cold loop” technique*		
Feasibility	0.931	0.854
	95% CI: 0.824–0.991	95% CI: 0.754–0.932
	*I* ^2^: 91.8%, *p* < 0.001	*I* ^2^ = 68.6%, *p*=0.041
	Begg's risk of bias: 0, *p*=0.750	Begg and Egger's risk of bias cannot be calculated (too few strata)
	Egger's risk of bias: 4.870, *p*=0.435	
Complication rate	0.0156	0.0285
	95% CI 0.0050–0.0318	95% CI: 0.0115–0.0530
	*I* ^2^: 32.4%, *p*=0.218	*I* ^2^ = 0%, *p*=0.779
	Begg's risk of bias 0.667, *p*=0.333	Begg's and Egger's risk of bias cannot be calculated (too few strata)
	Egger's risk of bias 0.434, *p*=0.454	

Sensitivity analyses confirmed the aforementioned overall proportions. The results are provided according to subgroups (Figure 2). The overall results in each subgroup are the weighted rate of feasibility and complications.

**Table 5:** Complications occurred (good-quality series).

	Complications (crude numbers)	Highest number reported	Highest rate reported
Hemorrhagic complications	21	5 (Hamidouche, Bipolar arm and Myosure® arm) [46]	14.7% (Hamidouche, Myosure® arm) [46]
Uterine perforations	15	3 (Fernandez) [84]	4.1% (Hamidouche, Bipolar arm) [46]
Intravasations	8	4 (Fernandez) [84]	2.5% (Hart) [47]
Infective complications	2	2 (Fernandez) [84]	1.1% (Fernandez) [84]
Cervical injuries/false routes	12	7 (Mazzon) [17]	4.8% (Mavrelos, GnRh arm) [66]
Tubal damages	—	—	—
Bowel injuries	2	1 (Mavrelos, Placebo arm) [66]	0.5% (Mavrelos, Placebo arm) [66]
		1 (Bernard) [28]	
Vaginal tear	1	1 (Mazzon) [17]	0.08% (Mazzon) [17]

Complications are reported as crude numbers, highest number reported, and highest rate reported.
